# The Identification of Loci for Immune Traits in Chickens Using a Genome-Wide Association Study

**DOI:** 10.1371/journal.pone.0117269

**Published:** 2015-03-30

**Authors:** Lei Zhang, Peng Li, Ranran Liu, Maiqing Zheng, Yan Sun, Dan Wu, Yaodong Hu, Jie Wen, Guiping Zhao

**Affiliations:** 1 Institute of Animal Science, Chinese Academy of Agricultural Sciences, Beijing, P.R. China; 2 Key Laboratory of Farm Animal Genetic Resources and Germplasm Innovation, Ministry of Agriculture, P.R. China, Beijing, P.R. China; University of Bologna, ITALY

## Abstract

The genetic improvement of disease resistance in poultry continues to be a challenge. To identify candidate genes and loci responsible for these traits, genome-wide association studies using the chicken 60k high density single nucleotide polymorphism (SNP) array for six immune traits, total serum immunoglobulin Y (IgY) level, numbers of, and the ratio of heterophils and lymphocytes, and antibody responses against Avian Influenza Virus (AIV) and Sheep Red Blood Cell (SRBC), were performed. RT-qPCR was used to quantify the relative expression of the identified candidate genes. Nine significantly associated SNPs (*P* < 2.81E-06) and 30 SNPs reaching the suggestively significant level (*P* < 5.62E-05) were identified. Five of the 10 SNPs that were suggestively associated with the antibody response to SRBC were located within or close to previously reported QTL regions. Fifteen SNPs reached a suggestive significance level for AIV antibody titer and seven were found on the sex chromosome Z. Seven suggestive markers involving five different SNPs were identified for the numbers of heterophils and lymphocytes, and the heterophil/lymphocyte ratio. Nine significant SNPs, all on chromosome 16, were significantly associated with serum total IgY concentration, and the five most significant were located within a narrow region spanning 6.4kb to 253.4kb (*P* = 1.20E-14 to 5.33E-08). After testing expression of five candidate genes (*IL4I1*, *CD1b*, *GNB2L1*, *TRIM27* and *ZNF692*) located in this region, changes in *IL4I1*, *CD1b* transcripts were consistent with the concentrations of IgY, while abundances of *TRIM27* and *ZNF692* showed reciprocal changes to those of IgY concentrations. This study has revealed 39 SNPs associated with six immune traits (total serum IgY level, numbers of, and the ratio of heterophils and lymphocytes, and antibody responses against AIV and SRBC) in Beijing-You chickens. The narrow region spanning 247kb on chromosome 16 is an important QTL for serum total IgY concentration. Five candidate genes related to IgY level validated here are novel and may play critical roles in the modulation of immune responses. Potentially useful candidate SNPs for marker-assisted selection for disease resistance are identified. It is highly likely that these candidate genes play roles in various aspects of the immune response in chickens.

## Introduction

Great effort has been expended globally to understand and genetically improve disease resistance in domestic animals [1–4]. Immune capacity associated with specific diseases may be useful indicators for indirect selection for general disease resistance, because such traits can be evaluated and quantified in live animals [5–9].

Immunological characteristics such as antibody titers have been shown to be heritable in poultry [10–11], indicating the possibility of discover loci or genes related to immune or disease resistance traits. Previous studies have found immune traits located in several chromosomal regions in chickens by microsatellite markers [10–12], and quantitative trait loci (QTLs) have been reported to be linked to the immune traits on chicken (Gallus gallus) chromosomes (GGA) 2, 3, 4, 5, 9, 13, 16, 18, 19, 22 and Z [13–21]. Only a few causative genes, however, have been identified because of low map resolution [22].

More recently, genome wide association studies (GWASs) have become one of the most commonly used strategies for identifying genes for complex traits in humans, as well as in animals [22]. In chickens, some major loci associated with resistance to Marek’s disease [23] and immune response to Newcastle disease virus and infectious bronchitis virus were identified by GWASs [9, 22]. Despite these studies, there is still limited information about the multiple immune traits that underly the full immune response at the genome-wide level in chickens.

This study aimed to identify major genomic regions (loci) and candidate genes associated with the immune response using GWAS for an array of important immune traits, including total serum concentrations of immunoglobulin Y (IgY), numbers of, and the ratio of heterophils and lymphocytes (H/L), and antibody responses against Avian Influenza Virus (AIV) and Sheep Red Blood Cells (SRBC) in chickens. This approach may offer valuable information for understanding the molecular mechanisms of the regulation of immune traits and facilitate the application of marker-assisted selection in breeding programs for disease resistance in chickens.

## Results and Discussion

### Genome-wide association analysis

**Fig 1:**
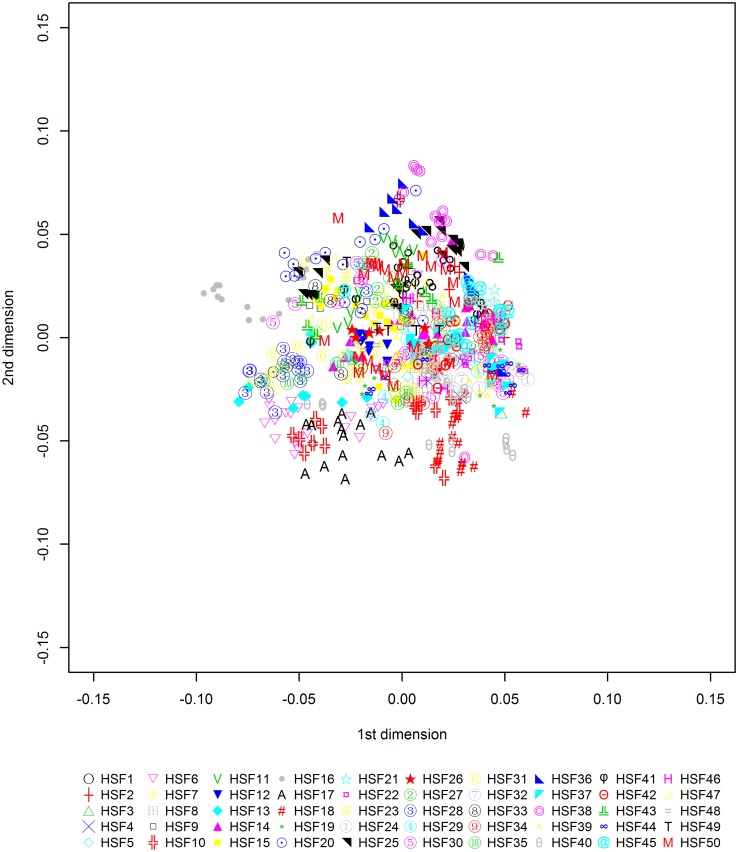
Sample structure evaluated by the first two MDS dimensions. HSF indicates half-sibling family.

A total of 7,175 independent SNP markers were obtained with multidimensional scaling (MDS) analysis of these SNPs using the first two principal components (Fig. 1) indicating that chickens within each half-sibling family were clustered together. No obvious population substructure was detected. The analytical method was as described previously [24] and resulted in the elimination of approximately 13,506 SNPs from further analysis. The distribution of SNPs after this quality control is summarized in Table 1. All raw genotypes are available from the Dryad Repository (doi:10.5061/dryad.8m4r5).

**Table 1:** Distribution of SNPs on each chromosome after quality control.

Chromosome	Physical length (Mb)^c^	No. SNPs	Marker density (kb/SNP)
1	200.9	6,876	29.2
2	154.8	5,172	29.9
3	113.6	3,925	28.9
4	94.2	3,155	29.9
5	62.2	2,096	29.7
6	37.4	1,613	23.2
7	38.3	1,761	21.7
8	30.6	1,329	23
9	25.5	1,162	21.9
10	22.5	1,259	17.9
11	21.9	1,163	18.8
12	20.5	1,314	15.6
13	18.9	1,068	17.7
14	15.8	970	16.3
15	12.9	947	13.6
16	0.43	20	21.5
17	11.1	831	13.4
18	10.9	803	13.6
19	9.8	792	12.4
20	13.9	1,428	9.7
21	6.9	771	8.9
22	3.9	269	14.5
23	6	587	10.2
24	6.4	653	9.8
25	2	167	12
26	5.1	632	8.1
27	4.8	430	11.2
28	4.5	542	8.3
LGE22C19W28_E50C23^a^	0.89	98	9.1
LGE64^a^	0.049	1	49
Z	74.6	1,737	42.9
0^b^	—	559	—
Total	1031.3	44,130	23.4

^a^ Two linkage groups.

^b^ These SNPs are not mapped to any chromosome.

^c^ The physical length of the chromosome was based on the genome build Gallus_gallus-2.1(May, 2006).

**Fig 2:**
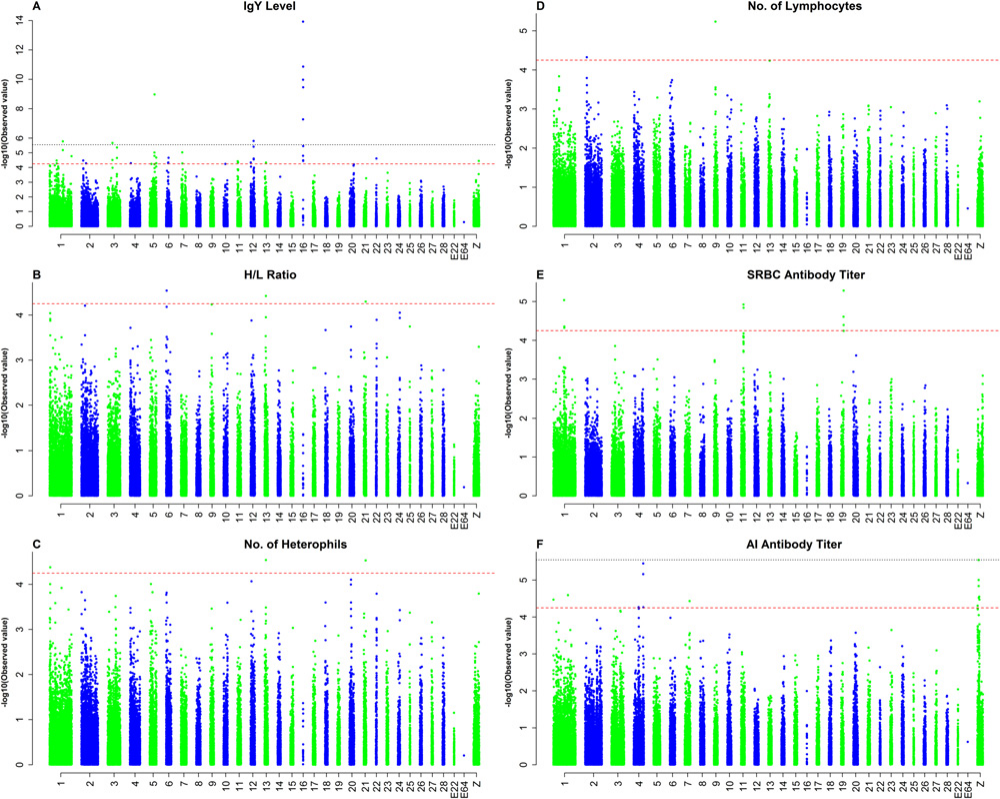
Manhattan plots showing association of all SNPs with six immune traits. SNPs are plotted on the x-axis according to their position on each chromosome against association with these traits on the y-axis (shown as-log10 *p*-value). The red dashed line indicates suggestive genome-wise significance (*p*-value = 5.62E-05), and the black dashed line shows genome-wise 5% significance with a p-value threshold of 2.81E-06. Fig. 2-A, 2-B, 2-C, 2-D, 2-E and 2-F refer to plots for total serum IgY level, H/L ratio, number of Heterophils, number of Lymphocytes, SRBC antibody titer and AI antibody titer, respectively.

The descriptive statistics for six immune traits in the 720 Beijing-You chickens used for the present GWAS are shown in Table 2. The numbers of genome-wide significant SNPs detected for the six immune traits, and the details of these significant SNPs, including their positions in the genome, the nearest known genes and the *P*-values, are given in Table 3 and Table 4. The global view of *P*-values (in terms of—log_10_ (*P*-value)) for all SNP markers of each trait is represented by a Manhattan plot shown in Fig. 2. The genome-wide association analysis in this study identified 39 SNPs, consisting of nine significantly associated SNPs and 30 suggestively associated SNPs.

**Table 2:** Descriptive statistics of the six immune traits.

Trait	Mean	Standard Deviation	Coefficient of Variation (%)
H/L Ratio	0.39	0.21	53.84
No. H	25.11	10.94	43.56
No. L	70.33	10.51	14.94
AIV Ab Titer（log2）	9.22	1.7	18.44
IgG Level (ng/ul)	538.59	279.91	51.97
SRBC Ab Titer（log2）	4.24	1.21	28.54

**Table 3:** Genome-wise 5% significant SNPs associated with total serum IgY level.

Trait	Chromosome	SNP ID	Position (bp)	Nearest Gene^a^	*P*-Value
IgY Level	16	Gga_rs16057130	6471	*CD1b* & *CD1c* ^b^	1.20E-14
	16	Gga_rs14738106	253412	2.9kb D *B-G* ^c^	1.38E-11
	16	Gga_rs15788101	110880	GNB2L1	1.08E-10
	16	GGaluGA111792	164922	LAO	3.50E-10
	16	Gga_rs15788237	59984	BMA1	5.33E-08
	5	GGaluGA285957	46399742	39.7kb U *TTC7B* ^d^	1.08E-09
	12	Gga_rs15661138	14395118	no gene	1.56E-06
	1	Gga_rs15399236	121689702	EIF2S3	1.66E-06
	3	Gga_rs16261002	44438665	40.1kb D *RNASET2*	2.14E-06

^a^ Genes located around 500kb upstream and downstream of the SNP were searched.

^b^Gene name alone indicates the SNP is within the gene.

^c^D indicates that the SNP is downstream of the gene.

^d^U indicates that the SNP is upstream of the gene.

These definitions are also used in Table 4.

**Table 4:** Suggestively Associated SNPs for five immune traits.

Trait	Chromosome	SNP ID	Position (bp)	Nearest Gene	*P-* Value
AIV Ab Titer	1	Gga_rs13937877	133493971	234kb D *SLC25A6*	2.54E-05
	1	Gga_rs15995401	111556	28kb D *HCLS1*	3.37E-05
	1	Gga_rs15995401	111556	28kb D *HCLS1*	3.37E-05
	4	GGaluGA269205	88150596	48kb U *FGFRL1*	3.53E-06
	4	GGaluGA269230	88264974	FGFRL1	6.88E-06
	4	GGaluGA255778	44000064	357kb U *AGA*	5.41E-05
	4	GGaluGA269388	88690383	24kb U *IMMT*	5.46E-05
	7	Gga_rs14626540	32964405	no gene	3.69E-05
	Z	GGaluGA348626	17004336	no gene	2.86E-06
	Z	Gga_rs16098994	17002454	no gene	9.90E-06
	Z	Gga_rs16099280	17569998	no gene	1.45E-05
	Z	Gga_rs16099440	17736692	no gene	3.00E-05
	Z	Gga_rs14778781	30762177	200kb D *TYRP1*	3.36E-05
	Z	Gga_rs14066816	3078791	no gene	4.92E-05
	Z	Gga_rs15648827	3147673	no gene	4.92E-05
SRBC Ab	1	Gga_rs13905265	95938436	68kb D *GPR89B*	9.15E-06
Titer	1	Gga_rs13907209	98680358	no gene	4.41E-05
	1	Gga_rs13907411	98949817	no gene	4.78E-05
	11	Gga_rs14025779	13991523	no gene	1.19E-05
	11	Gga_rs15619912	14576402	no gene	1.45E-05
	19	GGaluGA128939	9229902	48kb D *iNOS*	5.21E-06
	19	GGaluGA128957	9272295	1kb U *TNFAIP1*	5.21E-06
	19	Gga_rs15050742	9306653	21kb D *TNFAIP1*	5.21E-06
	19	Gga_rs10725789	9162804	iNOS	2.46E-05
	19	Gga_rs13576070	9265157	8kb U *TNFAIP1*	4.02E-05
H/L Ratio	6	Gga_rs14562554	4 673 503	559kb D *GHITM*	2.87E-05
	13	Gga_rs14051163	7 248 634	192kb D *GABRA6*	3.78E-05
	21	Gga_rs16743572	5 008 458	21kb D *DNAJC16*	5.03E-05
No. H	13	Gga_rs14051163	7 248 634	192kb D *GABRA6*	2.89E-05
	21	Gga_rs16743572	5 008 458	21kb D *DNAJC16*	2.94E-05
No. L	2	GGaluGA134321	14 283 920	11kb U *ZEB1*	4.73E-05
	9	Gga_rs15950814	10 984 983	PID1	5.82E-06

### Serum Concentrations of IgY at d 80

Five SNPs, associated with the serum total IgY level, were clustered within a 0.26 Mb region on chromosome 16 (S1 Table). One SNP, rs14738106, was located 2.9 kb downstream of the *B-G* gene (within the MHC region). Four of the SNPs (rs16057130, rs14738106, rs15788101 and rs15788237) were located within *CD1b* and *CD1c* genes, an intron of *GNB2L1* gene, an intron of *IL4I1* gene and an intron of *BMA1* gene, respectively. A QTL for titres of Natural antibodies (NA) was detected at the SNP rs15788216 on chromosome 16 in chickens [14–15], near the SNP rs15788237 found here.

The chicken MHC, which is composed of the B and Y complexes [25] and plays a central role in the immune response, has also been mapped to this NOR (Nucleolus Organizing Region, cluster of genes for ribosomal RNA 18S, 5.8S and 28S)-bearing microchromosome 16 [26].

*CD1b* and *CD1c* encode two members of the *CD1* family, which are structurally similar to the *MHC* class I antigen and have been shown to present lipid antigens for recognition by T lymphocytes [27]. The expression of the *IL4I1* gene can be induced by interleukin 4 in B cells. The IL4I1 protein has L-phenylalanine oxidase activity, and oxidative deamination of phenylalanine by IL4I1 produces H_2_O_2_, and functions in the innate immune defenses of vertebrates. Previous studies have revealed that the IL4I1 protein inhibits T-cell proliferation with a preference for memory T cells, and H_2_O_2_ mediates this inhibition [28–29].

In the current study, significant SNPs on GGA1, 3, 5 and 12 were also identified that related to serum total IgY level. Those were not located within the previously known QTLs for immune traits, for example, five QTLs associated with humoral innate immune response in chickens (NA titers binding LPS, located on GGA5, GGA9, GGA14, GGA18, and GGAZ) were recently validated in a cross between Green-Legged Partridge-like and White Leghorn [19, 21]. The reason for the discrepancy might result from the different chicken populations being studied.

### Heterophils and Lymphocytes

The heterophil to lymphocyte (H/L) ratio in the peripheral blood of chickens has been widely accepted as a reliable and accurate physiological indicator of the stress response in chickens [30–31]. The number of lymphocytes decrease and the number of heterophils increase in response to stressors such as high temperature, hypothermy, excessive NH_3_, bacterial infection, *inter alia* [32–33]. The ratio, and individual numbers of heterophils and lymphocytes are highly heritable traits, with heritability estimates exceeding 0.5 [34], suggesting that good responses to selection should be possible.

There were three SNPs detected as being suggestively associated with the H/L ratio (S2 Table), two of which were simultaneously associated with the number of heterophils (S3 Table). One SNP (rs14562554) was mapped to 559kb downstream of the growth hormone inducible transmembrane protein (*GHITM*) gene on chromosome 6. The GHITM protein is part of the interferon signaling system that includes Janus kinases and their downstream target STAT proteins [35]. One SNP at 7.2Mb on chromosome 13 is located 192kb downstream of gamma-aminobutyric acid receptor subunit alpha-6 (*GABRA6)*. This gene is associated with increased production of cortisol and increased blood pressure in response to psychological stress [36]. The rs16743572 SNP is located downstream of DnaJ homolog, subfamily C, member 16 (*DNAJC16*) in chromosome 21; the gene is a member of the *DnaJ/Hsp40* (heat shock protein 40) family. These proteins are molecular chaperones induced in response to cellular stress [37] and have been preserved throughout evolution, functioning in protein translation, folding, unfolding, translocation, and degradation, primarily by stimulating the ATPase activity of Hsp70s chaperone proteins [38]. Human Hsp40s have an immune-modulatory effect on T cells and cytokine secretion in rheumatoid arthritis patients [39].

There were two SNPs, rs15950814 and GGaluGA134321, that were suggestively associated with the number of lymphocytes (S4 Table). The former is located within phosphotyrosine interaction domain containing 1 (*PID1*), also called *NYGGF4*, on chromosome 9, which encodes a protein possessing a phosphotyrosine interaction domain (PID). Mouse and human *NYGGF4* are involved in the regulation of proliferation of adipocyte precursors [40]. The gene is more widely expressed in chicken tissues than in those of mice and humans [41]. Proteins with PID are widely involved in physiological processes, such as neural development, immune function, homeostasis and cell growth [42], which indicates that the chicken *NYGGF4* homolog possibly possesses multiple functions. The GGaluGA134321 SNP, is located 11kb downstream of the zinc finger E-box binding homeobox 1 (*ZEB1*) gene on chromosome 2. In human and mouse studies, ZEB1 is a transcriptional repressor and plays an important role in regulating lymphoid differentiation [43]. It has been implicated in the regulation of other important genes in the immune system such as the Ig heavy chain enhancer, *CD4*, *GATA-3*, *α7-integrin* and *IL-2* [44–45]. It has been shown that *ZEB1*-deficient mice have poorly developed thymuses and significant decreases in their T cell population [46].

### Antibody responses to SRBC

The direct response to selection on antibody titer to SRBC challenge is accompanied by correlated responses in production and disease-related traits [13, 16]. The heritability of anti-SRBC titer in chickens is moderate (0.20 to 0.25), and the trait responds to divergent selection [47].

In this study, of special interest, five SNPs in a narrow region (9.16Mb—9.31Mb) on chromosome 19 were suggestively associated with antibody titer against SRBC (S5 Table). The rs10725789 and GGaluGA128939 are located within and 48 kb downstream of the inducible nitric oxide synthase (*iNOS*) gene. The expression of *iNOS* is regulated by cytokines such as IFN-γ, IL-1β, IL-6, TNF-α, and iNOS-derived nitric oxide (NO) is well known to play important roles in infection and immunity [48]. The rs15050742, rs13576070 and GGaluGA128957 SNPs are located 21kb downstream, 1kb and 8kb upstream of the tumor necrosis factor, alpha-induced protein 1 (TNFAIP1). The TNFAIP1 protein is a TNF-α and IL-6 inducible protein. Research in humans and rats indicate that the protein may have roles in DNA synthesis and repair and in apoptosis [49]. Such results refine the detail of this genomic region and lead to more conclusive identification of the contributing loci involved and the putative causal mutations.

In addition, three SNPs on chromosome 1 were suggestively associated with anti-SRBC titer (S5 Table), and rs13905265 is 68 kb downstream of G protein-coupled receptor 89B (*GPR89B*), but no known genes were found within 500 kb of the other two SNPs. A QTL has been detected in the vicinity of this region in a F2 cross of two lines divergently selected for SRBC antibody responses [16]. Two SNPs of suggestive significance at 13.9 Mb and 14.6 Mb are consistent with a genomic region identified in a previous study in chickens [50]. There are several other genomic regions previously identified as QTLs for SRBC antibody response in chickens, including those on GGA2, GGA3, GGA5, GGA11, GGA16, GGA23 and GGA24 [13, 16–19, 21, 50–51].

### Antibody responses to AIV

Two SNPs on chromosome 1 were suggestively associated with AIV antibody titer (S6 Table). One SNP, rs13937877 is located 234 kb downstream of solute carrier family 25, member 6 (*SLC25A6*). The present study found that the region at about 100 Mb from the proximal end of chicken chromosome 1, including the *ROBO1* (Roundabout, axon guidance receptor, homolog 1) and *ROBO2* (homolog 2) genes, has a strong effect on the antibody response to the NDV in chickens [22]. One SNP rs14527240 was located in the *SPARC*-related modular calcium-binding 1 *(SMOC1)* gene on GGA5 playing an important role in MD tumorigenesis [23]. GGA1 also had the most significantly associated SNPs on the antibody level against infectious bronchitis virus (IBV), but a genomic region containing a cluster of 13 beta-defensin *(GAL1–13)* and interleukin-17F genes on GGA3 probably plays an important role in the immune response against IBV [9]. The *SLC25A6* gene encodes adenine nucleotide translocase 3 (ANT3), an enzyme which exchanges ATP and ADP through the mitochondrial membrane. Mitochondrial *ANT3* is regulated by IL-4 and IFN-γ via STAT-dependent pathways [52]. Considering the critical role of mitochondrial ANTs in energy metabolism and apoptosis, ANT3 regulation by IL-4 and IFN-γ may have a functional implication in cytokine-mediated T cell survival [53].

One SNP (rs15995401) is located 28 kb downstream of hematopoietic cell-specific Lyn substrate 1 (*HCLS1*). HCLS1 is an intracellular protein expressed mainly in hematopoietic cells. It was reported to be an important actin regulator at the T-cell immunologic synapse and also to influence numerous functions of natural killer cells [54].

Eight SNPs on chromosome Z were suggestively novel and specific associated with AIV antibody titer in this study (S6 Table). One SNP (rs14778781) is located within multiple PDZ domain protein (*MPDZ*) which is involved in a diverse range of pathways that maintain cell integrity and function [55]. No known genes were found within 500kb of the remaining seven SNPs. These studies concerning immune responses against NDV, IBV and AIV add to basic knowledge of host attributes potentially relevant to improved methods for controlling outbreaks of viral diseases.

### Relative expression of candidate genes in spleen

The present GWAS verified that the important immune-related locus was located on chromosome 16 [9, 22]. In this study, five of the significant SNPs for total serum IgY level were located within a narrow 247 kb region on chromosome 16. On this basis, the genes within this region were selected for further expression analysis, including *CD1b*, *IL4I1* (Interleukin 4-induced 1), *TRIM27* (tripartite motif-containing 27), *GNB2L1* (guanine nucleotide binding protein, beta polypeptide 2-like 1), and *ZNF692* (zinc finger protein 692).

Compared with SRBC, *Salmonella enteritidis* is more potent in inducing production of serum antibodies (IgY) in the infected chickens. For just that reason, it was used here to validate the association between total serum IgY level and the corresponding candidate genes identified in this study.

**Fig 3:**
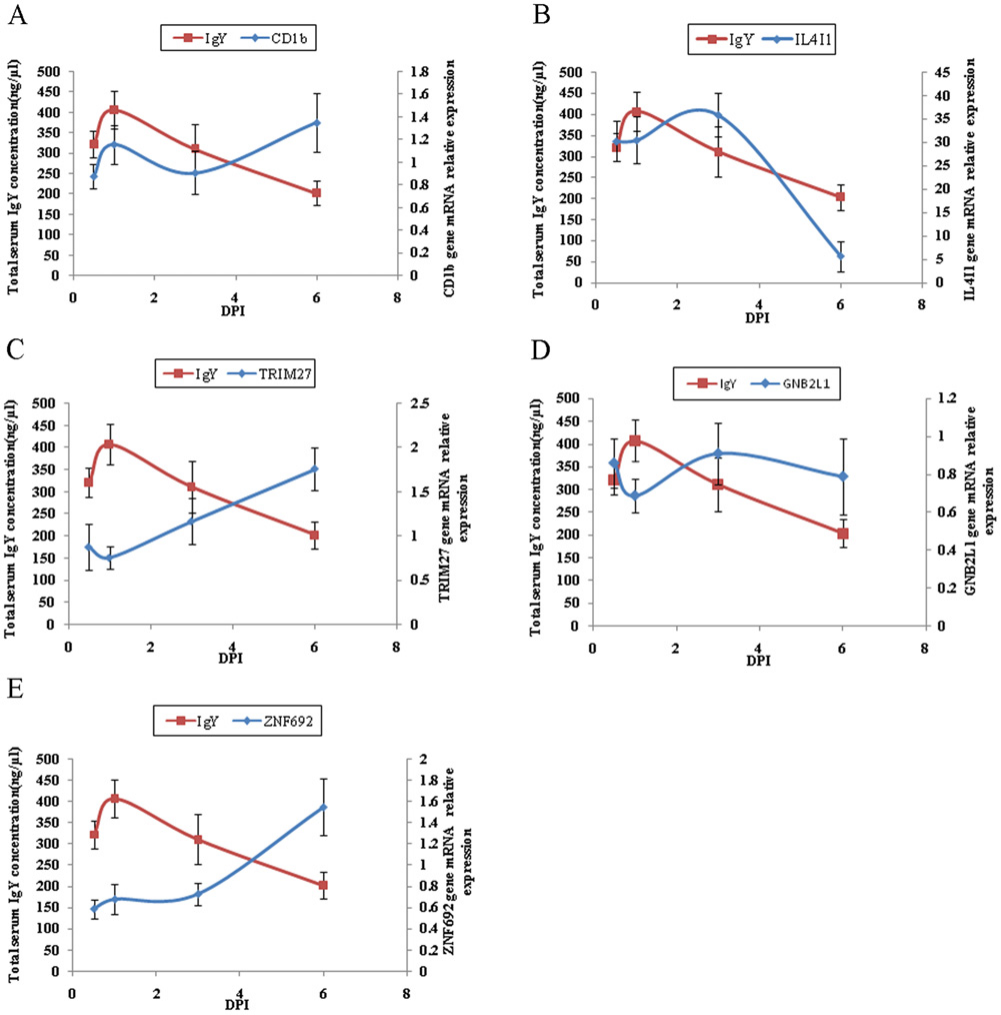
Changes in relative transcript abundance of five genes in spleen at 12h, 24h, 3 or 6 days post-infection (DPI) and total serum IgY concentration. The relative expression of *CD1b* (A), *IL4I1* (B), *TRIM27* (C), *GNB2L1* (D), and *ZNF692* (E) was measured in spleen at different times post-infection with *Salmonella enteritidis* (10^8^ CFU) (n = 6). Serum concentrations of total IgY of SE-infected and uninfected birds were determined by chicken ELISA kits. Results were expressed as the mean fold-change in gene expression at each time point, from triplicate analyses, using the non-infected control sample at 0.5 dpi as the calibrator (assigned an expression level of 1). Each point-in-time represents the mean ± S.D. of 6 birds. There were barely detectable or no changes in relative expression of the five candidate genes and total IgY concentration in un-challenged controls (data not shown).

The relative expression of those five candidate genes near associated signals from the GWAS analysis was determined by RT-qPCR in chickens with and without challenge with *Salmonella enteritidis*. In this study, the changes across days in expression of *IL4I1* and *CD1b* followed a similar pattern to the concentrations of IgY, while changes in *TRIM27* (alternatively named RET finger protein, RFP) and *ZNF692* expression were the reverse (mirror-image) of concentrations of IgY (Fig. 3).

Chickens possess two *CD1* genes designated *chCD1–1* and *chCD1–2* that are closely linked to the MHC locus on chromosome 16. Both genes are expressed in lymphoid tissues as evidenced by Northern analysis and PCR [56]. The spleen was examined here because it is the major lymphoid organ in birds and has previously been shown to have high level expression of *CD1* [57].

*IL4I1* modulates the number of antitumor cytotoxic T cells *in vivo* and affects their capacity to secrete IFN-γ. The expression of *IL4I1* is restricted to lymphoid tissues, with the highest levels found in lymph nodes and spleen [58]. In the present study, the expression of *CD1b* and *IL4I1* was up-regulated in spleens of infected birds.

*TRIM27* is a member of the TRIM superfamily and exhibits the classical PRY-SPRY domain C-terminal of the RBCC motif. *TRIM27* is highly expressed in mouse spleen and thymus [59] and in hematopoietic cells [60]. Roles for *TRIM27* in transcriptional repression [60], the negative regulation of NF-κB and in IFN-signalling pathways, apoptosis and in cell cycle regulation have been reported [61], indicating its involvement the control of multiple cellular processes. These findings account for the present demonstration that the abundance of *TRIM27* showed reciprocal changes to those of IgY concentrations.

The genes *ZNF692* and *GNB2L1* relate to the *MHC* genes, but little is known of their precise function in chickens. The changes in relative expression and relationship to changes in IgY after infection, support their having some role, probably negative regulatory, in the magnitude of the immune response.

## Conclusions

This study has revealed 39 SNPs associated with six immune traits (total serum IgY level, numbers of, and the ratio of heterophils and lymphocytes, and antibody responses against AIV and SRBC) in Beijing-You chickens. Five of the significant SNPs for total serum IgY level were located within a narrow 247 kb region on chromosome 16. Some SNPs were located within or close to the previously reported QTL regions. Five candidate genes related to IgY level by GWAS, and validated here by experimental challenge, are novel and may play critical roles in the modulation of immune responses. It is highly likely that these candidate genes play an important role in the immune response. Findings herein lay an important foundation for further identifying the immunity-related genes and provide useful information for the molecular mechanisms underlying disease-resistance traits in chickens.

## Materials and Methods

### Ethics Statement

All of the animal experiments were conducted in accordance with the Guidelines for Experimental Animals established by the Ministry of Science and Technology (Beijing, China). Animal experiments were approved by the Science Research Department (in charge of animal welfare issue) of the Institute of Animal Sciences, CAAS (Beijing, China).

### Animal resource

Beijing-You chickens (a Chinese indigenous breed) from the national conservation farm, Institute of Animal Sciences, CAAS, were used. A total of 728 male offspring from 50 sire families were produced in one hatch. The birds were reared in stair-step cages under standard conditions of humidity, temperature, and ventilation, and had access to water and feed *ad libitum*. All chickens received the conventional inoculation regimen (S7 Table), including vaccination against H5N1 avian influenza virus (AIV) on d 17.

### Phenotypic measurements

All blood samples were collected from the brachial vein by venipuncture. At d 49, chickens were injected intramuscularly in the leg with 1 ml of a 0.25% suspension of SRBC. Sera were isolated from blood collected on d 55 (6d post-injection) for immediate determination of anti-SRBC titers by hemagglutination [62] using 96-well plates and the same preparation of SRBC as used for the immunization. Antibody titers were expressed as the log2 of the reciprocal of the highest dilution giving complete agglutination. On d 62, blood smears were prepared from each chicken, stained with May-Grunwald and Giemsa stains and differentially counted [63]. One hundred leukocytes, including granular (heterophils, eosinophils, and basophils) and nongranular (lymphocytes and monocytes), were counted on one slide from each bird, and the heterophil to lymphocyte ratio (H/L) was calculated. Concentrations of IgY and anti-AIV titers were measured in serum collected on d 80. A double antibody sandwich enzyme-linked immunosorbent assay (ELISA) was used for IgY (R&D Systerm, Minneapolis, USA) and a hemagglutination-inhibition diagnostic test was used for titering anti-AIV. The descriptive statistics of phenotypic values of all traits were analyzed using the MEANS procedure in SAS 9.2 software (SAS Institute Inc., Cary, NC).

### Genotyping and quality control

Genomic DNA (gDNA) was extracted from blood samples using phenol-chloroform. The DNA samples were genotyped using the Illumina 60K chicken SNP Beadchip (DNA LandMarks Inc., Saint-Jean-sur-Richelieu, Quebec, Canada). The intensity files generated were processed with the GenomeStudio GT module with default settings [64].

Quality control was performed by PLINK 1.07 [65] and eight chickens were excluded because of high genotyping missing rate (> 95%). SNPs failing to meet any one of the following filtering criteria were excluded: call rate > 90%; minimum minor allele frequency (MAF) > 0.03; *P*-value for Hardy-Weinberg equilibrium test > 1×10^–6^.

### Statistical analysis

To evaluate if population substructure existed among the individuals, classical MDS, as implemented by PLINK 1.07 [65], was performed. The procedures were as follows: first, to ensure uncorrected LD did not distort the analysis, all autosomal SNPs passing the quality control were subjected to LD-based pruning, thus the remaining SNPs within a given window size of 25 had pairwise r^2^ < 0.2; second, the pairwise IBS distance among the 720 individuals was calculated using the remaining 7,175 SNPs; finally, the first two MDS dimensions were extracted via the “MDS-plot” command and visualized in R v3.0.1 (www.r-project.org).

Association analyses were conducted to test the additive effects of allele dosage by PLINK, using the linear regression model. The fitted equation was:

Yij=b0+∑bk−1fk+b50Xij+εij

Where: *Y*_*ij*_ is the phenotypic value of a given immune trait for individual *i* in the *j* ^th^ sire family, *b*_*0*_ is the intercept, *b*_*k-1*_, *b*_*50*_ is the regression coefficient, *f* is the dummy variable for the sire family, *k* is an integer ranging from 2 to 50, *X*_*ij*_ denote the genotype at the given SNP, and *ε*_*ij*_ is independent, normally distributed random residual, with mean 0 and constant variance. For the family variable with 50 categories, 49 binary dummy variables *f* (coded 0/1), created by the dummy-coding option within PLINK, were fitted in the model.

Given the correlation between SNPs in linkage disequilibrium, the strict Bonferroni adjustment appears to be overly conservative [66]. The sum of independent LD blocks plus singleton markers was used to define the number of independent statistical comparisons [67], as described in [68]. Finally, 17,794 independent tests were used to determine the *P*-value thresholds, including 5% genome-wide significance (2.81E-6, 0.05/17,794) and suggestive association (5.62E-5, 1/17,794). A global view of the *p*-value results for the six traits from the association analysis was created by the “gap” package [66] in R v3.0.1 (www.r-project.org).

### Relative expression of candidate genes identified by the GWAS

**Birds and Treatment.** Twelve-day-old Beijing-You chickens were from the same conservation stock and were reared as described earlier, in separate cages. Birds were confirmed to be free of *Salmonella* by culturing fecal samples in buffered peptone water (BPW) overnight with shaking at 150 rpm and spreading on brilliant green agar containing 100 mg/ml nalidixic acid (37°C, 18–24 h). Chickens were randomly divided into 2 groups of 30 each (*Salmonella enteritidis* challenge and un-challenged controls). Birds in the challenged group were injected intramuscularly into the leg with 0.5 ml of *SE* bacterial suspension containing 10^8^ CFU and the control group was given 0.5 ml saline. Six chickens from each group were randomly chosen, and sacrificed at 12h, 24h, 3 or 6 days post-infection. Blood was collected, allowed to clot, and serum was stored. The spleen was rapidly removed, snap-frozen in liquid nitrogen, and stored at-80°C for mRNA extraction.

**Serological test.** Concentrations of total IgY in all sampled birds were determined in triplicate by ELISA, as described earlier.

**mRNA abundance of candidate genes by RT-qPCR.** Total RNA was isolated from each spleen sample with the RNAsimple Total RNA kit (TIANGEN BIOTECH, Beijing, China) and first-strand cDNA was synthesised from 2 μg total RNA using the Promega Reverse Transcription Kit (Promega, Beijing, China), according to the manufacturer’s instructions. Power SYBR Green PCR Master Mix (Applied Biosystems, USA) was used to quantify mRNA abundance of the genes by Reverse Transcription-Quantitative PCR (RT-qPCR) with an ABI 7500 Real-time Detection System (Applied Biosystems, USA). The primers were designed with Primer Premier 5.0 from the GenBank sequences (Table 5). The PCR amplification was performed, as previously described [24]. To determine fold-changes in gene expression, the comparative CT method was used [69], calculated as 2^-ΔΔCT^. Results were expressed as the mean fold-change in gene expression at each time, from triplicate analyses, using the non-infected control sample at 0.5dpi as the calibrator (assigned an expression level of 1). All analyses were performed using Student’s t-test and data are presented as means ± S.D.

**Table 5:** Primers used for the RT-qPCR.

Gene	Primer	（5’-3’）Primers sequence	Product/bp	GenBank No.
*β-actin*	F	GAGAAATTGTGCGTGACATCA	152	NM_205518.1
	R	CCTGAACCTCTCATTGCCA		
*CD1b*	F	CATTGCTCTTGGGCAACGTC	113	NM_001024582
	R	TGGCCGGCAGATGTTTAAGT		
*IL4I1*	F	GAAGCACGTTGGCAGGAAAG	138	NC_006103.3
	R	AACCTGCACGAAGTCGGAAT		
*GNB2L1*	F	GCAGCAACCCCATCATTGTC	188	NM_001004378
	R	ATTCAGGTCCCACAGCATGG		
*TRIM27*	F	GCCGAGCTGGAGAAGAAACT	247	NM-0010099354.1
	R	CCAGCACGCAGAAGGAGTAA		
*ZNF692*	F	GGAAAGCAGCGAAACGTGAA	116	NM_001099356.1
	R	GGTCTTCTGGTGGACGTGTT		

## Supporting Information

S1 Table Raw linear regression result for Serum IgY Level. The raw results from the linear regression analysis by PLINK. Its basic format is: CHR, Chromosome; SNP, SNP identifier; BP, Physical position (base-pair); A1, Tested allele (minor allele by default); TEST, Code for the test (the additive effects of SNPs); NMISS, Number of non-missing individuals included in analysis; BETA, Regression coefficient (the direction of BETA represents the effect of each extra minor allele); STAT, Coefficient t-statistic; P, Asymptotic *p*-value for t-statistic. The same abbreviations are used in the following tables.
(XLSX)

S2 Table Raw linear regression result for the Ratio of H and L. (XLSX)

S3 Table Raw linear regression result for the Number of H. (XLSX)

S4 Table Raw linear regression result for the Number of L. (XLSX)

S5 Table Raw linear regression result for SRBC Antibody Titer. (XLSX)

S6 Table Raw linear regression result for AI Antibody Titer. (XLSX)

S7 Table The convention of vaccination program in this study. (DOCX)
