# Supplementary material for: The Identification of Loci for Immune Traits in Chickens Using a Genome-Wide Association Study
Source: PLoS One. 2015 Mar 30;10(3):e0117269. doi: 10.1371/journal.pone.0117269 (PMC4378930; doi:10.1371/journal.pone.0117269)
Supplement: S7 Table — (DOCX) [file pone.0117269.s007.docx]

| **Age** | **vaccine** | **Doses** | **Pattern** | **Manufacturer** |
| --- | --- | --- | --- | --- |
| 1 day | VAXXITEK HVT+IBD | 0.3ml | subcutaneous injection | Merial, China |
| 7 day | NB BLEN(Lasota+B48) |  | ocular/nasal drops | Merial, China |
| 17 day | AI killed vaccine (Re4+Re6) | 0.3ml | intramuscular injection | QYH Biotech Co., Ltd., China |
| 21 day | ND Killed vaccine(Lasota) | 0.3ml | intramuscular injection | Merial, China |
| 23 day | NB BLEN(Lasota+B48) |  | ocular/nasal drops | Merial, China |
| 28 day | AI killed vaccine (Re4+Re6) | 0.3ml | intramuscular injection | QYH Biotech Co., Ltd., China |
| 35 day | Pox live vaccine |  | by puncture of wing membrane | Merial, China |

**Table S7. The convention of vaccination program in this study.**
